# Evaluating plasma ferritin and suPAR as treatable traits for critically ill patients with COVID-19 treated with anakinra: an exploratory analysis of the immune modulation domain of the REMAP-CAP randomised clinical trial

**DOI:** 10.1186/s13054-026-06311-3

**Published:** 2026-10-01

**Authors:** Andrew J. Boyle, Elizabeth G. Ryan, Lindsay R. Berry, Scott Berry, Lennie Derde, Patrick R. Lawler, Elizabeth Lorenzi, Cecilia M. O’Kane, Manu Shankar-Hari, Frank van de Veerdonk, Anthony C. Gordon, Daniel F. McAuley

**Affiliations:** 1 Wellcome-Wolfson Institute for Experimental Medicine, Queen’s University Belfast, 97 Lisburn Road, Belfast, Northern Ireland; 2 Regional Intensive Care Unit, Royal Victoria Hospital, Grosvenor Road, Belfast, Northern Ireland; 3 School of Public Health and Preventive Medicine, Monash University, Melbourne, Australia; 4 Berry Consultants LLC, Austin, TX USA; 5 Intensive Care Center, University Medical Center Utrecht, Utrecht, the Netherlands; 6 Julius Center for Health Sciences and Primary Care, University Medical Center Utrecht, Utrecht, The Netherlands; 7 European Research Alliance for Infectious Diseases (Ecraid) Foundation, Utrecht, the Netherlands; 8 Department of Medicine, McGill University Health Centre, Montreal, Canada; 9 Centre for Critical Illness Research, School of Immunology and Microbial Sciences, King’s College London, London, UK; 10 Department of Internal Medicine, Radboud Center for Infectious Diseases, Radboud University Medical Center, Nijmegen, The Netherlands; 11 Division of Anaesthetics, Pain Medicine & Intensive Care, Imperial College London, London, UK

**Keywords:** COVID-19, REMAP-CAP, Ferritin, suPAR

## Abstract

**Background:**

Identifying treatable traits in critically ill patients offers potential to enable a precision medicine approach to treatment. The aim of this exploratory analysis of the REMAP-CAP immune modulation domain was to evaluate whether plasma ferritin or soluble urokinase plasminogen activator receptor (suPAR) represent treatable traits for anakinra in critically ill patients with coronavirus disease (COVID-19).

**Methods:**

An exploratory analysis of patients randomised within the COVID-19 immune modulation domain of the REMAP-CAP trial was conducted. Adults within 24 h of receiving respiratory or cardiovascular organ support in an ICU with suspected or microbiologically confirmed COVID-19 were included. Plasma ferritin was measured at study sites, and plasma suPAR measured in stored plasma samples. The primary outcome was organ support-free days (OSFD) until day 21. Analyses were performed using a Bayesian cumulative logistic regression model, and results are presented as the posterior median odds ratio (OR), along with 95% credible intervals (CrI), for anakinra being superior to control.

**Results:**

In ferritin analyses, 1243 patients were included. Patients with increasing ferritin levels had numerically fewer OSFDs. The posterior probabilities of anakinra having benefit (OR > 1) were 64%, 71%, and 21% across increasing ferritin terciles, compared to 53% in analyses that did not adjust for ferritin levels. 145 patients were included in the suPAR analyses. Patients with high baseline plasma suPAR (≥ 6 ng/ml; *n* = 85) had numerically fewer OSFDs than patients with low plasma suPAR (-1.0 (-1.0, 9.0) vs. 13.0 (-1.0, 17.0)). The posterior probabilities of anakinra having benefit (OR > 1) were 94.8% (OR 2.28 [0.85–6.06]) in patients with low baseline suPAR, and 38.6% (OR 0.88 [0.39–2.00]) in patients with high baseline suPAR.

**Conclusion:**

Plasma ferritin does not identify patients with COVID-19 who benefit from treatment with anakinra, whilst anakinra may be beneficial in patients with low, rather than high, baseline plasma suPAR. These hypothesis-generating findings contrast with the results of clinical trials in non-critically ill patients with COVID-19.

**Trial registration:**

NCT02735707. Date of trial registration: 06/04/2016

**Supplementary Information:**

The online version contains supplementary material available at https://doi.org/10.1186/s13054-026-06311-3.

## Background

It is increasingly recognised that patient heterogeneity restricts the ability to identify effective therapeutics in the critically ill [1]. Within clinical syndromes, such as sepsis or acute respiratory distress syndrome (ARDS), re-analyses of clinical trials that originally showed no treatment effect have identified patient subgroups that have a differential treatment response [2–9]. The Randomized Embedded Multifactorial Adaptive Platform for Community Acquired Pneumonia (REMAP-CAP) trial is an international, adaptive platform trial evaluating multiple treatments for patients with severe pneumonia in both pandemic and non-pandemic settings (NCT02735707). During the coronavirus disease (COVID-19) pandemic, eligible patients enrolled in REMAP-CAP were randomised to receive anakinra, an interleukin (IL)-1 receptor antagonist, as part of the COVID-19 immune modulation domain. In the original analysis of patients enrolled into this domain, anakinra was found to be ineffective [10].

We hypothesised that there were subgroups of patients with severe COVID-19 for whom there is a heterogenous treatment effect for anakinra. In a re-analysis of a clinical trial of patients with sepsis without COVID-19, anakinra was associated with reduced 28-day mortality in a subgroup of patients with clinical features of macrophage activation syndrome (MAS) [11]. Recently, a clinical trial of patients with sepsis has demonstrated that hyperferritinaemia, as a marker of MAS, identifies patients who benefit from treatment with anakinra [12]. Hyperferritinaemia is independently associated with mortality in patients with ARDS [13]. Although MAS is uncommon in critically ill patients with COVID-19, they do have a dysregulated host immune response that is characterised by IL-1-driven inflammation [14], similar to patients with MAS. Therefore, it is plausible that ferritin may identify critically ill patients with COVID-19 who may benefit from treatment with anakinra.

Anakinra has also been demonstrated to improve clinical outcomes in non-critically ill patients with COVID-19 and high plasma soluble urokinase plasminogen activator receptor (suPAR) [15]. In critically ill patients, high plasma suPAR is associated with an increased risk of acute kidney injury [16] and ARDS [17]. However, it is unknown whether plasma suPAR identifies critically ill patients with COVID-19 who have an increased risk of mortality, or if it represents a treatable trait for anakinra in this patient group.

The aims of this exploratory analysis were to evaluate if the efficacy of anakinra varies by baseline plasma ferritin or suPAR concentration in critically ill patients with COVID-19.

## Methods

### Trial design

We performed a post-hoc exploratory analysis of the previously reported REMAP-CAP clinical trial [18]. REMAP-CAP is an ongoing international, adaptive platform trial designed to determine the best treatment strategies for patients with severe community-acquired pneumonia, in both pandemic and non-pandemic settings. Eligible patients are randomised to one or more interventions across multiple domains. Details of trial design, governance and approvals have been described previously [10, 19]. The trial was conducted in accordance with Good Clinical Practice guidelines and the principles of the Declaration of Helsinki.

### Patients

Adults aged ≥ 18 years, within 24 h of receiving respiratory or cardiovascular organ support in an intensive care unit (ICU) with suspected or microbiologically confirmed COVID-19 who were recruited to the COVID-19 immune modulation domain of REMAP-CAP were included in this analysis if they had baseline plasma ferritin or suPAR values available for analysis. Respiratory organ support was defined as invasive or non-invasive mechanical ventilation, including via high flow nasal cannula if the flow rate was > 30 L/min and the fraction of inspired oxygen was > 0.4. Cardiovascular organ support was defined as the intravenous infusion of any vasopressor or inotrope. Exclusion criteria within REMAP-CAP included imminent death and prior participation in the trial within the previous 90 days prior to randomisation. The COVID-19 Immune Modulation Domain included five interventions: anakinra, two IL-6 receptor antagonists (tocilizumab, sarilumab), interferon-β1a, and control (no immune modulation). Additional exclusion criteria specific to the COVID-19 Immune Modulation Domain and the interventions are included in supplementary file 1.

### Procedures

Details of drug dosing and administration have previously been reported [10]. Participants were randomised via a centralised computer program with allocation ratios initially dependent on the number of interventions available at each site. There was variation in allocation ratios based on regular adaptive analyses to increase the randomisation probabilities for more promising treatments (response-adaptive randomisation). Investigators at each site selected a priori at least two of the interventions to which participants could be randomised; and initially one of these interventions had to be control.

Plasma ferritin was measured at study sites by each institution’s laboratory. Plasma suPAR was measured using enzyme-linked immunoassay in stored samples from patients in the immune-modulation domain of REMAP-CAP who were co-enrolled in the convalescent plasma domain of REMAP-CAP, during which plasma samples were collected for future analysis.

### Outcomes

The primary outcome for this analysis was organ support-free days (OSFD) until day 21, an ordinal endpoint with in-hospital mortality coded as the worst outcome (‐1) to ensure that death was treated as a worse outcome than no days free of organ support. Organ support was defined as it was for the inclusion criteria. Survival to hospital discharge (censored at 90 days) was evaluated as a secondary outcome.

### Statistics

REMAP-CAP uses a Bayesian adaptive platform trial design with no maximum sample size; adaptive analyses are performed on a regular time basis and randomisation continues within a domain until certain pre-defined statistical triggers have been met. There was no a priori defined sample size for this exploratory analysis. The study population was subdivided for the two biomarker analyses:

1. Ferritin population

All patients who met the above inclusion criteria and had available baseline plasma ferritin values were studied (primary ferritin population) in analyses of the effect of baseline plasma ferritin on anakinra. In analyses of the primary ferritin population, patients from each of the four immune modulation domain treatment arms (control, anakinra, IFN-β1a and IL-6 receptor antagonists) were included, but only results pertaining to anakinra (vs. control) are presented. Data from the other treatment arms was not pooled. As not all patients were eligible for all treatments, and therefore to exclude bias that may have arisen from factors influencing treatment eligibility, a sensitivity analysis was undertaken in only those patients with baseline plasma ferritin values who were eligible for both anakinra and control and were randomised to either of these treatment arms (secondary ferritin population).

2. suPAR population

To assess if the efficacy of anakinra varies by baseline plasma suPAR, patients meeting the above eligibility criteria who were randomised to anakinra or control and had baseline plasma suPAR values were evaluated (suPAR population). These patients were only recruited from within the United Kingdom because this is the only region where the convalescent plasma domain of REMAP-CAP was running and samples were collected as part of that domain.

The analysis model for OSFD was a Bayesian cumulative logistic model. Survival to hospital discharge (dichotomous outcome) was modelled using Bayesian logistic regressions. The statistical models included adjustments for the following covariates: site (random effect), age group (≤ 39, 40–49, 50–59, 60–69, 70–79, 80+), sex, and time period of enrolment (12-week epochs; to account for potential temporal effects such as rapid changes in clinical care and outcomes during the COVID-19 pandemic) [20]. The statistical models for the primary ferritin population also included an effect for each intervention (compared to control). Prior distributions for treatment effects were centred on no effect (standard normal neutral priors).

Separate analyses were performed to explore baseline ferritin and suPAR as potential effect modifiers for anakinra, and several different modelling strategies were used to investigate the relationship between the biomarker (ferritin or suPAR), outcome (OSFD or survival), and treatment effect (of anakinra) (see Table S1, supplementary file, for a description of the statistical models). For both biomarkers, initial models were fit within each analysis population to estimate the overall anakinra treatment effect (compared to control) without incorporation of baseline ferritin or suPAR into the analysis (these are referred to as the “reference models”).

In the absence of a defined plasma ferritin threshold that is associated with outcome in severe COVID-19, the primary method used in ferritin analyses was based on baseline ferritin tercile groups in the primary ferritin population. Analyses were undertaken estimating the overall effect of anakinra with baseline ferritin terciles included as covariates in the model. Next, analyses were run with subgroups defined based on ferritin tercile. In these analyses, separate effects of anakinra were estimated within each tercile group. These analyses were then repeated in the ferritin sensitivity analysis population. To enable more flexible modelling, additional analyses were performed by defining subgroups according to baseline ferritin quintiles. Analyses were first performed to adjust for covariate effects across the 5 ferritin subgroups with a first-order normal dynamic linear model (NDLM) prior (to smooth the effects), and then separate anakinra treatment effects were estimated within each subgroup using an NDLM. Finally, the relationship between ferritin and the outcome was modelled more flexibly by fitting a spline (penalized cubic order B-spline with 5-knots) to the log transformed baseline ferritin values. Models were fit with a spline of log baseline ferritin (covariate adjustment) and to estimate differential treatment effects of anakinra across the log baseline ferritin levels (using splines). These latter models (using quintiles and splines) were supportive analyses that were only fit to the primary ferritin analysis population due to sample size limitations and are presented in the supplementary material.

In contrast, analyses were conducted for the suPAR population using two subgroups defined as suPAR < 6 ng/ml and ≥ 6 ng/ml, respectively. This threshold for dichotomisation was selected because existing data supports it as having both prognostic and predictive enrichment properties in COVID-19 [15, 21]. Additional methods were not deployed due to the smaller sample size of the suPAR population. In the suPAR analyses, the models were fit to estimate the overall effect of anakinra with suPAR group included as a covariate in the model, and then to estimate differential treatment effects of anakinra in each of the suPAR groups. Further details of these models are provided in Table S1.

The models were fit using a Markov Chain Monte Carlo algorithm (30,000 draws), which calculated the posterior distribution of the treatment and covariate effects. Only participants with complete data were included in the analysis populations; no imputation was performed. Results from each of these models (for both outcomes) are reported as odds-ratios (OR) where OR > 1 implies clinical benefit (relative to the reference group). The posterior median OR, along with 95% credible intervals (CrI), mean and standard deviation of the estimates are reported. The models also provide the posterior probability of anakinra being superior to control (Pr(OR > 1)). Given the exploratory nature of this analysis, no thresholds were applied to evaluate statistical significance of these models.

The analyses were conducted in accordance with a pre-specified Statistical Analysis Plan (SAP) (supplementary file 2). Due to small sample sizes for some analysis populations, modifications were made to the pre-specified analyses in the SAP to improve model stability. Limited analyses were performed for suPAR due to moderate sample sizes in the analysis population.

All merging and summarisation of data was done using the R statistical computing environment (v 4.4.0). The Bayesian models were fitted using Stan (v 2.32.2) via the rstan package (v2.32.6). Further details of all analyses are provided, and prespecified analyses are listed in the supplemental material.

### Results

The COVID-19, Immune Modulation Domain of REMAP-CAP began patient recruitment on 25 March 2020. The control arm closed on 19 November 2020 when the statistical trigger for superiority of tocilizumab compared with control was met. Otherwise, enrolment to receive tocilizumab, sarilumab, interferon-β1a and anakinra continued until 9 April 2021, following which the domain closed to further recruitment [10]. The first patient eligible for this exploratory analysis was enrolled on 23rd April 2020 (Figure S1, supplementary file 1). Flow diagrams demonstrating how the analysis populations were derived are presented in the supplementary file (Figure S2 and S3).

### Baseline characteristics of patients included in ferritin analyses

The baseline characteristics for the primary ferritin population are presented in Table 1. There were 1243 patients enrolled in the REMAP-CAP COVID-19 Immune Modulation Domain who had plasma ferritin measured (primary ferritin population). Of these patients, 706 (57%) were eligible, and 203 (16%) were randomised to anakinra. Of 456 (37%) patients eligible to receive control, 205 (16%) were randomised to control. The primary ferritin population was similar to the patient group who did not have baseline plasma ferritin measurements available (Table S2). The baseline characteristics of patients included in the ferritin secondary population (only patients eligible for both anakinra and control with baseline plasma ferritin measurement; *n* = 48) were similar to those of the primary ferritin population (Table S3).

**Table 1:** Baseline characteristics for primary ferritin and suPAR analysis populations

		Primary ferritin population ^a,b^ (n=1243)	suPAR population ^a,c^ (n=145)
Treatment allocation			
Control		205 (16.5)	78 (53.8)
Anakinra		203 (16.3)	67 (46.2)
IL-6 receptor antagonist		823 (66.2)	N/a
Interferon-β1a		12 (1.0)	N/a
Age in years, median (IQR)		61.0 (52.0, 69.0)	61.0 (53.0, 70.0)
Male sex		857 (68.9)	106 (73.1)
Race / Ethnicity^d^			
Asian		159/961 (16.5)	25/133 (18.8)
Black		40/961 (4.2)	7/133 (5.3)
Mixed		17/961 (1.8)	2/133 (1.5)
White		689/961 (71.7)	93/133 (69.9)
Other		56/961 (5.8)	6/133 (4.5)
Body-mass index ^e^– median (IQR)		30.8 (26.8, 35.5) (n=1152)	31.2 (27.1, 34.3) (n=127)
APACHE II score ^f^ – median (IQR)		13.0 (8.0, 19.0) (n=1229)	12.0 (7.0, 19.2) (n=144)
Confirmed SARS-CoV-2 infection		1065/1136 (93.8)	136/140 (97.1)
Preexisting condition ^g ^			
Diabetes		379 (30.5)	51 (35.2)
Respiratory disease		279 (22.4)	27 (18.6)
Asthma/COPD		236 (19.0)	24 (16.6)
Other		56 (4.5)	4 (2.8)
Kidney disease		82/1152 (7.1)	10/132 (7.6)
Severe cardiovascular disease		114/1242 (9.2)	17/144 (11.8)
Any immunosuppressive condition		35 (2.8)	4 (2.8)
Cancer		15 (1.2)	2 (1.4)
Chronic immunosuppressive therapy		14 (1.1)	0 (0.0)
Other		14 (1.1)	2 (1.4)
Liver cirrhosis/failure		2/1242 (0.2)	0/144 (0.0)
Time to enrollment – median (IQR)			
From hospital admission (days)		1.3 (0.9, 3.1)	1.1 (0.8, 2.6)
From ICU admission (hours)		13.5 (6.4, 19.4)	15.0 (7.5, 20.1)
Acute respiratory support			
Invasive mechanical ventilation		410 (33.0)	42 (29.0)
Noninvasive ventilation only		511 (41.1)	68 (46.9)
High-flow nasal cannula		319 (25.7)	33 (22.8)
None / supplemental oxygen		3 (0.2)	2 (1.4)
ECMO		1/1233 (0.1)	1 (0.7)
Vasopressor support		235 (18.9)	26 (17.9)
PaO_2_ / FiO_2_ – median (IQR)		112.0 (87.0, 151.0) (n=1169)	111.5 (88.8, 169.2) (n=128)
Laboratory values – median (IQR) ^h^			
C reactive protein (µg/mL)		124.0 (70.5, 197.8) (n=1090)	124.0 (77.0, 206.0) (n=117)
D-dimer (µg/L)		1007.0 (510.0, 2412.0) (n=1017)	1006.5 (474.5, 1620.0) (n=82)
Plasma ferritin (µg/L)		1005.0 (548.5, 1688.5)	914.0 (458.0, 1787.0) (n=85)
Plasma suPAR (ng/mL)		6.9 (5.0-8.7) (n=85)	6.7 (5.3-8.7)
Received therapies at randomisation^i^			
Corticosteroids		1153/1242 (92.8)	136 (93.8)
Remdesivir		483/1241 (38.9)	75 (51.7)
Country			
United Kingdom		1037 (83.4)	145 (100.0)
Netherlands		124 (10.0)	0 (0.0)
Saudi Arabia		68 (5.5)	0 (0.0)
Ireland		10 (0.8)	0 (0.0)
Other		4 (0.3)	0 (0.0)

Data displayed as n(%) unless otherwise stated

a. Patients aged ≥18 years, within 24 hours of receiving respiratory or cardiovascular organ support in an intensive care unit (ICU) with suspected or microbiologically confirmed COVID-19 who were

b. recruited to the COVID-19 immune modulation domain of REMAP-CAP and whom had available baseline plasma ferritin result

c. randomised to anakinra or control within the COVID-19 immune modulation domain of REMAP-CAP and whom had available baseline plasma suPAR results

d. Data collection was not approved in Canada and continental Europe. 'Other' includes 'declined' and 'multiple’. Participants (or their surrogates) self-reported their race/ ethnicity via fixed categories appropriate to their region

e. Body-mass index is the weight in kilograms divided by the square of the height in meters

f. Acute Physiology and Chronic Health Evaluation II scores range 0 to 71, with higher scores indicating greater severity of illness

g. Kidney disease was determined from the most recent serum creatinine level prior to this hospital admission, except in patients who were receiving dialysis. Abnormal kidney function was defined as a creatinine level of 130 μmol/L or greater (1.5mg/dL) for males or 100 μmol/L or greater (1.1 mg/dL) for females not previously receiving dialysis. Cardiovascular disease was defined as New York Heart Association class IV symptoms. Immunosuppression was defined by the receipt of recent chemotherapy, radiation, high-dose or long-term steroid treatment, or presence of immunosuppressive disease

h. Laboratory results available when captured for clinical care

i. Within 48hr of randomisation

### Ferritin analyses

In the primary ferritin population, the median baseline plasma ferritin was 1005.0 (IQR 548.5, 1688.5) µg/L, whilst in the secondary ferritin population median baseline plasma ferritin was 1046 (679.8, 1696.0) µg/L (Table 1 and Table S3). The unadjusted outcome data for both populations are presented in Table 2, S4 and visual representation of OSFDs in both populations are presented in Figures S4 and S5. In the primary ferritin population, there were numerically fewer OSFDs, and numerically more hospital deaths, across increasing ferritin terciles (Tables S5 and S6). Similar patterns were observed when baseline plasma ferritin was examined by quintiles (Figure S6), and in the secondary ferritin population (Tables S5 and S6).

**Table 2:** Outcome data

	Primary ferritin population ^a,b^	suPAR population
	(N=1243)	(N=145) ^a, c^
OSFD – median (IQR)		
Overall population	5.0 (-1.0, 16.0)	0.0 (-1.0, 15.0)
By treatment group:		
Control	4.0 (-1.0, 16.0)	0.0 (-1.0, 14.0)
Anakinra	0.0 (-1.0, 16.0)	0.0 (-1.0, 16.0)
IL-6 receptor antagonist	8.0 (-1.0, 17.0)	N/a
Interferon-β1a	3.5 (-0.2, 12.2)	N/a
In-hospital mortality – n (%)		
Overall population	417 (33.5)	62 (42.8)
By treatment group:		
Control	72 (35.1)	29 (37.2)
Anakinra	77 (37.9)	33 (49.3)
IL-6 receptor antagonist	265 (32.2)	N/a
Interferon-β1a	3 (25.0)	N/a
28-day mortality – n/N (%)		
Overall population	347/1218 (28.5)	50/145 (34.5)
By treatment group:		
Control	61/203 (30.0)	23/78 (29.5)
Anakinra	61/195 (31.3)	27/67 (40.3)
IL-6 receptor antagonist	224/809 (27.7)	N/a
Interferon-β1a	1/11 (9.1)	N/a
Length of hospital stay – median (95% CI) ^d^		
Overall population	29 (26, 34)	38 (28, N/a)
By treatment group:		
Control	29 (24, 44)	36.5 (25, N/a)
Anakinra	35 (25, 56)	46.0 (23, N/a)
IL-6 receptor antagonist	28 (24, 33)	N/a
Interferon-β1a	33 (16, N/a)	N/a

a. Patients aged ≥18 years, within 24 hours of receiving respiratory or cardiovascular organ support in an intensive care unit (ICU) with suspected or microbiologically confirmed COVID-19 who were

b. recruited to the COVID-19 immune modulation domain of REMAP-CAP and whom had available baseline plasma ferritin results

c. randomised to anakinra or control within the COVID-19 immune modulation domain of REMAP-CAP and whom had available baseline plasma suPAR results

d. N/a values for the upper 95% CI reflect a small sample size too small to calculate an upper margin of error

**Fig. 1:**
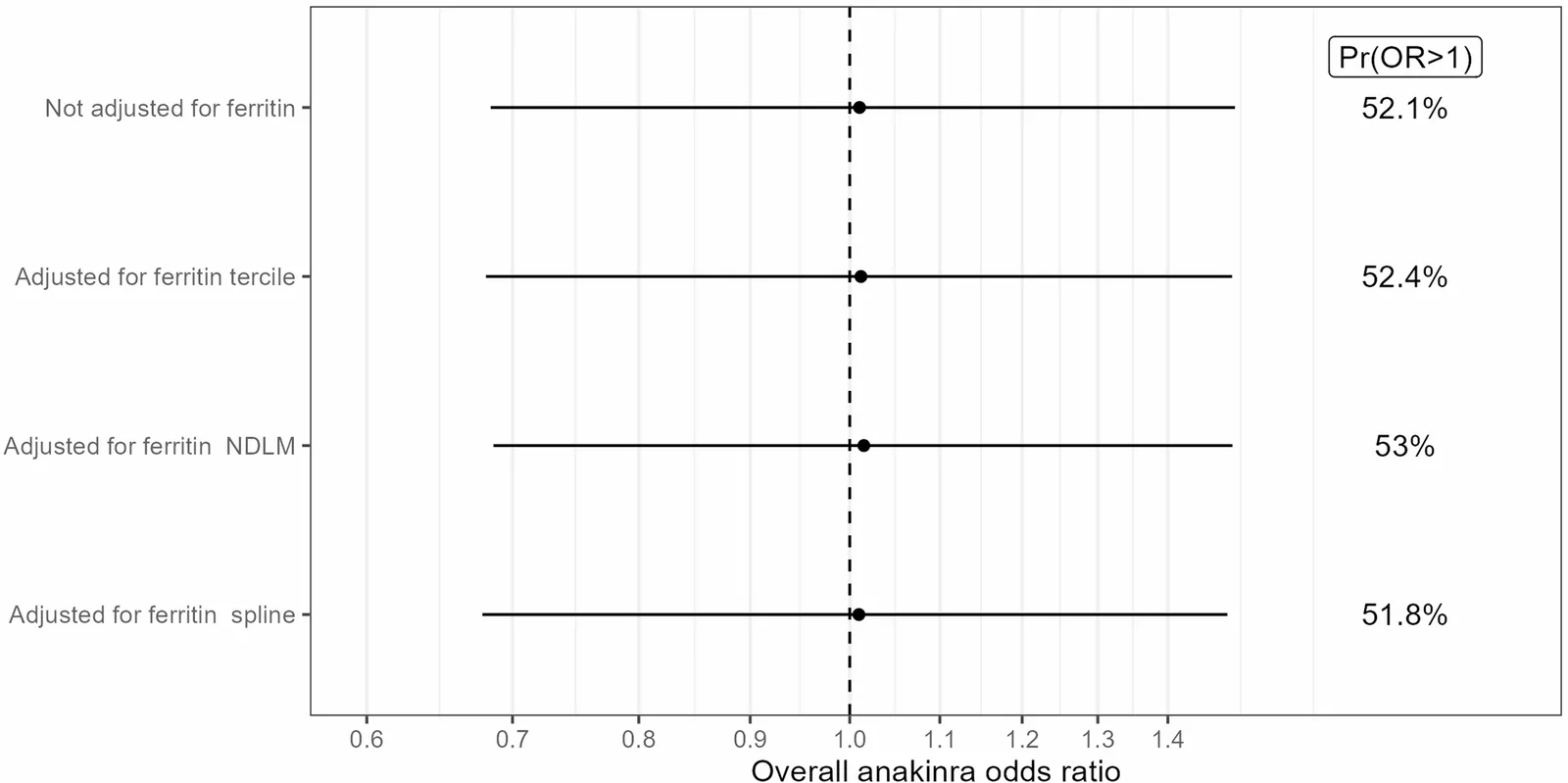
Forest plot showing overall anakinra effect on OSFDs in population 1 with and without adjustment for ferritin. Forest plot of the overall anakinra treatment effect (measured in odds ratio) on OSFDs with and without adjustment for ferritin. PR(OR > 1): probability of odds ratio > 1 for anakinra having benefit compared to control for OSFDs

In multivariable analysis of OSFDs in the primary ferritin population, after adjustment for age, sex, time-period, and site, the median odds ratio for anakinra was 1.01 [95% CrI 0.68–1.50], with a posterior probability of benefit (OR > 1 compared with control) of 52.1% (Reference model) (Table S7). Adjustment for baseline ferritin levels in the model, irrespective of the method used, had little impact on the anakinra treatment effect estimates or posterior probabilities of benefit (Fig. 1). The highest posterior probability of benefit on OSFDs for a treatment interaction with anakinra was for the second ferritin tercile (median OR 1.16 [95% CrI 0.68–1.99]; posterior probability of benefit (OR > 1 compared with control) 71.1%) (Table 3). These findings were similar when ferritin quintiles and cubic splines were analysed (Table S8 and Figure S7a and S7b). In the secondary ferritin population, whilst results were similar, the highest posterior probability of benefit on OSFDs for a treatment interaction with anakinra was for the lowest ferritin tercile (median OR 1.71 [95% CrI 0.39–7.60]; posterior probability of benefit (OR > 1 compared with control) 76.0% (Tables S9-11).

**Table 3:** Multivariable regression for OSFD in primary ferritin analysis population including anakinra-ferritin treatment interaction

Variable	Median OR	CrI	Mean OR	Pr(OR>1)
			(standard deviation)	
Anakinra (Ferritin Tercile 1)	1.1	(0.64, 1.87)	1.14 (0.32)	0.635
Anakinra (Ferritin Tercile 2)	1.16	(0.68, 1.99)	1.21 (0.34)	0.711
Anakinra (Ferritin Tercile 3)	0.79	(0.45, 1.39)	0.83 (0.24)	0.206
Baseline Ferritin Tercile 2	0.87	(0.66, 1.14)	0.88 (0.12)	
Baseline Ferritin Tercile 3	0.81	(0.61, 1.08)	0.82 (0.12)	
Age<39	4.8	(3.16, 7.31)	4.91 (1.07)	
Age 40-49	2.4	(1.74, 3.30)	2.43 (0.40)	
Age 50-59	2.23	(1.72, 2.91)	2.25 (0.31)	
Age 70-79	0.55	(0.40, 0.75)	0.56 (0.09)	
Age 80+	0.34	(0.18, 0.62)	0.36 (0.11)	
Female	1.28	(1.02, 1.61)	1.29 (0.15)	

Multivariable analysis of patients aged ≥18 years, within 24 hours of receiving respiratory or cardiovascular organ support in an intensive care unit (ICU) with suspected or microbiologically confirmed COVID-19 who were recruited to the COVID-19 immune modulation domain of REMAP-CAP and whom had available baseline plasma ferritin results

In multivariable analysis evaluating hospital mortality as the dependant outcome in the primary ferritin population, addition of ferritin to multivariable models did not substantially modify the treatment effect of anakinra on hospital mortality (Figure S8). Results were similar in the secondary ferritin population (Table S12).

### suPAR analyses

Baseline plasma suPAR was measured in 145 patients randomised to receive either anakinra (*n* = 67; 46%) or control (*n* = 78; 54%). The baseline characteristics for this population are presented in Table 1. Median baseline plasma suPAR concentration was 6.65 (IQR 5.27, 8.66) ng/ml (Table 1). The suPAR analysis population was comprised of only patients recruited within the UK, however this population was similar to patients randomised to anakinra or control in whom there was no plasma suPAR measurement (Table S13).

Unadjusted outcomes for this population are presented in Table 2 with a visual representation of OSFDs presented in Figure S9. When dichotomised by plasma suPAR, patients with high baseline plasma suPAR (≥ 6 ng/ml; *n* = 85) were more frequently allocated to anakinra, were numerically older and had both numerically fewer OSFDs (-1.0 (-1.0, 9.0) vs. 13.0 (-1.0, 17.0)) and numerically higher in-hospital mortality (54.1% vs. 26.7%) than patients with low plasma suPAR (Tables S14 and S15).

In multivariable analysis without adjustment for plasma suPAR, the median odds ratio for anakinra was 0.92 [0.48–1.76], generating a posterior probability for benefit of anakinra (OR > 1 compared to control) of 40.7% (reference model; Table S16). Adjustment for high plasma suPAR in multivariable modelling increased the odds ratio of a benefit with anakinra (median OR 1.33 [0.66–2.61]) with a numerically higher posterior probability for benefit (OR > 1 compared with control) (78.9% vs. 40.7%) (Table S17).

The median odds ratio for the anakinra interaction effect with baseline plasma suPAR on OSFDs was numerically higher with low suPAR (2.28 [0.85–6.06]) than with high suPAR (0.88 [0.39–2.00]), with posterior probability for benefit (OR > 1 compared with control) 94.8% and 38.6% respectively (Table 4).

**Table 4:** Multivariable model for OSFDs in suPAR population including anakinra-suPAR treatment interaction

Variable	Median OR [95% credible interval]	Mean OR (standard deviation)	Pr(OR > 1)
Anakinra (low baseline plasma suPAR)	2.28 [0.85, 6.06]	2.58 (1.40)	0.948
Anakinra (high baseline plasma suPAR)	0.88 [0.39, 2.00]	0.96 (0.42)	0.386
High baseline plasma suPAR	0.50 [0.23, 1.10]	0.54 (0.23)	
Age < 39	6.89 [2.11, 23.79]	8.42 (5.92)	
Age 40–49	1.98 [0.86, 4.70]	2.18 (1.00)	
Age 50–59	1.89 [0.91, 4.06]	2.04 (0.82)	
Age 70–79	0.59 [0.26, 1.35]	0.65 (0.29)	
Age 80+	0.59 [0.15, 2.10]	0.72 (0.52)	
Female	1.05 [0.53, 2.07]	1.12 (0.40)	

Multivariable analysis patients aged ≥ 18 years, within 24 h of receiving respiratory or cardiovascular organ support in an intensive care unit (ICU) with suspected or microbiologically confirmed COVID-19 who were randomised to anakinra or control within the COVID-19 immune modulation domain of REMAP-CAP and whom had available baseline plasma suPAR results

Findings were similar when hospital mortality was assessed as the dependant outcome (Tables S18-S20).

### Discussion

In this exploratory analysis of critically ill patients with COVID-19 who were enrolled within the COVID-19 Immune Modulation Domain of the REMAP-CAP trial, there are three key findings. First, clinical outcomes were worse in critically ill patients with COVID-19 who had higher plasma ferritin or high plasma suPAR (≥ 6 ng/ml) measured at randomisation. Second, baseline plasma ferritin concentration does not appear to guide the effectiveness of treatment with anakinra in critically ill patients with COVID-19. Finally, patients with low plasma suPAR have a numerically higher posterior probability of benefit from anakinra, compared with control, for both OSFDs and hospital mortality, than patients with high plasma suPAR. These findings should be considered hypothesis-generating.

High plasma ferritin is regarded as a feature of MAS [22], and in patients with sepsis and either hyperferritinaemia or clinical features of MAS, anakinra is associated with improved outcomes [11, 12]. Like MAS, cytokine release syndrome can be characterised by hyperferritinaemia, fever and raised inflammatory markers. However, in patients with COVID-19 and cytokine release syndrome (the definition of which was in part based on plasma ferritin concentration ≥ 700 µg/L), there was no treatment effect identified for anakinra [23]. In this exploratory analysis of REMAP-CAP, although clinical outcomes were numerically worse in patients with higher ferritin, there was no evidence of a differential response to anakinra according to ferritin levels. Although this exploratory analysis did not classify patients as having macrophage activation syndrome or cytokine release syndrome, either by ferritin concentration nor by clinical phenotype, the contrasting results between patients with COVID-19 and sepsis highlights the risks of translating findings between different groups of critically ill patients. The potential for similar subphenotypes to have different effects between parent clinical syndromes has recently been shown in critically ill patients with acute hypoxaemic respiratory failure. When classified using as either fulfilling criteria for hyper/hypoinflammatory subphenotypes, the subphenotypes were not as clearly associated with clinical outcomes in patients with non-ARDS acute hypoxaemic respiratory failure as they were in patients fulfilling ARDS diagnostic criteria [24].

As expected, patients with high baseline plasma suPAR (≥ 6 ng/ml) had numerically worse clinical outcomes. These results are in keeping with prior data that demonstrated that plasma suPAR can aid the identification of patients with COVID-19 who are likely to require critical care admission [21]. Anakinra has been shown to be of benefit in non-critically ill patients admitted to hospital with COVID-19 who have high plasma suPAR [15]. In contrast, in this analysis we demonstrate that critically ill patients with COVID-19 and low baseline plasma suPAR had a numerically higher probability of better outcomes after treatment with anakinra compared with control for both OSFD and hospital mortality than patients with high plasma suPAR. The reasons for the contrasting findings between critically ill and non-critically ill patients are unclear. Although the effect of anakinra in non-critically ill patients with low suPAR (< 6 ng/ml) is unknown, it is plausible that the benefit for anakinra is in preventing critical illness in patients with high suPAR. Alternatively, any potential benefit from anakinra may be overwhelmed by systemic illness severity in critically ill patients, which may be highest in patients with high plasma suPAR who are known to have greater organ dysfunction. In REMAP-CAP, suPAR levels were measured in stored plasma samples in contrast to SAVE-MORE where it was measured directly to guide patient inclusion in the trial. This may be important because differences in sample handing can influence suPAR measurement [25]. Whilst there was a large numerical difference in the treatment effect of anakinra between high/low baseline plasma suPAR that favoured low plasma suPAR, the 95% credible intervals for these interaction terms overlapped and in this small sample size these results may be exaggerated. Although the statistical interaction terms suggested that patients with low plasma suPAR may benefit most from treatment with anakinra, the finding that baseline plasma suPAR was higher in non-survivors is consistent with previous evaluations of critically ill patients [26, 27]. Although there may be differences in the cell source of circulating suPAR between patients based on illness severity [28], the biological mechanisms through which plasma suPAR enriches for an anakinra treatment effect are unknown, and therefore it is uncertain whether the findings in this analysis are biologically plausible. Much like the ferritin data, these findings highlight the importance of conducting well designed clinical trials in specific patient populations to avoid translation of treatment effects from one population into another in whom the effects have not been prospectively evaluated. Finally, these results re-demonstrate the need for further studies of the biological mechanisms that may link plasma suPAR and anakinra, and the role of predictive enrichment strategies, between distinct patient groups.

This analysis has several strengths in keeping with an international, randomised, clinical trial. However, there are some specific limitations to this exploratory analysis. First, most patients in the ferritin analysis, and all patients in the suPAR cohort, were recruited from within the United Kingdom. Although these patients were similar to broader cohorts of patients without available plasma samples, this limits the generalisability of these findings. Furthermore, plasma suPAR values were only available in patients who were co-enrolled in the convalescent plasma domain of REMAP-CAP, and therefore there is a risk of selection bias that may also limit generalisability. Second, there was a small number of patients eligible for inclusion in this analysis, thus limiting the certainty of many of these findings. Third, plasma ferritin was measured at each site, and therefore variation between the assays and platforms used to measure ferritin may have impacted the results [29, 30]. Finally, this was a post-hoc exploratory analysis, and although a pre-defined SAP was developed, this study was not prospectively powered to detect differences between patient populations, and these descriptive results should be considered exploratory.

## Conclusions

In summary, in this exploratory analysis of the immune modulation domain of the REMAP-CAP platform clinical trial, we found that plasma ferritin does not identify patients with COVID-19 who benefit from treatment with anakinra, whilst patients with low plasma suPAR have numerically better clinical outcomes and may have more benefit from treatment with anakinra than patients with high plasma suPAR. These hypothesis-generating findings contrast with the results of clinical trials in non-critically ill patients with COVID-19. Future studies may wish to further evaluate the role of plasma suPAR to provide predictive enrichment for anakinra.

## Supplementary Information

Below is the link to the electronic supplementary material.

Supplementary Material 1

## Data Availability

The datasets used may be made available from the REMAP-CAP investigators upon reasonable request to the corresponding author.

## Abbreviations

ARDS: Acute respiratory distress syndrome
COVID-19: Coronavirus disease
CrI: Credible intervals
ICU: Intensive care unit
IL: Interleukin
MAS: Macrophage activation syndrome
OR: Odds ratio
OSFD: Organ support free days
Pr: Posterior probability
SAP: Statistical analysis plan
suPAR: Soluble urokinase plasminogen activator receptor
REMAP-CAP: Randomized embedded multifactorial adaptive platform for community acquired pneumonia trial
