# Supplementary material for: Evaluating plasma ferritin and suPAR as treatable traits for critically ill patients with COVID-19 treated with anakinra: an exploratory analysis of the immune modulation domain of the REMAP-CAP randomised clinical trial
Source: Crit Care. 2026 Oct 1;30:506. doi: 10.1186/s13054-026-06311-3 (PMC13629143; doi:10.1186/s13054-026-06311-3)
Supplement: Supplementary file 1 [file 13054_2026_6311_MOESM1_ESM.docx]

**Evaluating plasma ferritin and suPAR as treatable traits for critically ill patients with COVID-19 treated with anakinra: an exploratory analysis of the Immune Modulation Domain of the REMAP-CAP randomised clinical trial**

Andrew J Boyle^1,2^, Elizabeth G Ryan^3^, Lindsay R Berry^4^, Scott Berry^4^, Lennie Derde^5,6,7^, Patrick R Lawler^8^, Elizabeth Lorenzi^4^, Cecilia M O’Kane^1^, Manu Shankar-Hari^9^, Frank van de Veerdonk^10^, Anthony C Gordon^11^, and Daniel F McAuley^1,2^ on behalf of the REMAP-CAP Immune Modulation Domain Investigators

[Platform trial exclusion criteria	3](#_Toc234409443)

[Immune modulation domain specific exclusion criteria	4](#_Toc234409444)

[Intervention-specific exclusion criteria	5](#_Toc234409445)

[Table S1: Description of statistical models	6](#_Toc234409446)

[Table S2: Baseline characteristics of patients in the Immune Modulation Domain of REMAP-CAP with severe COVID-19 dichotomised by availability of baseline ferritin measurement	9](#_Toc234409447)

[Table S3: Baseline characteristics of secondary ferritin population	12](#_Toc234409448)

[Table S4: Outcomes of secondary ferritin population	13](#_Toc234409449)

[Table S5: OSFDs stratified by ferritin tercile	14](#_Toc234409450)

[Table S6: In-hospital mortality stratified by ferritin tercile	15](#_Toc234409451)

[Table S7: Cumulative logistic regression model for OSFD in primary ferritin population (Reference model)	16](#_Toc234409452)

[Table S8: Cumulative logistic regression model for OSFD with ferritin quintiles-anakinra interaction terms in primary ferritin population	17](#_Toc234409453)

[Table S9: Cumulative logistic regression model for OSFD in secondary ferritin population (reference model)	18](#_Toc234409454)

[Table S10: Cumulative logistic regression model for OSFD with ferritin terciles as covariates in secondary ferritin population	19](#_Toc234409455)

[Table S11: Cumulative logistic regression model for OSFD with ferritin terciles-anakinra interaction terms in secondary ferritin population	20](#_Toc234409456)

[Table S12: Logistic regression model for hospital survival exploring differential treatment effect with ferritin terciles in secondary ferritin population	21](#_Toc234409457)

[Table S13: Baseline characteristics based on availability of baseline plasma suPAR	22](#_Toc234409458)

[Table S14: Baseline characteristics in suPAR population dichotomised by baseline plasma suPAR	25](#_Toc234409459)

[Table S15: OSFD and hospital mortality in suPAR population dichotomised by baseline plasma suPAR	26](#_Toc234409460)

[Table S16: Cumulative logistic regression model for OSFD in suPAR population (reference model)	27](#_Toc234409461)

[Table S17: Cumulative logistic regression model for OSFDs in suPAR analysis population with adjustment for high baseline plasma suPAR	28](#_Toc234409462)

[Table S18: Logistic regression model for hospital survival in suPAR population (reference model)	29](#_Toc234409463)

[Table S19: Logistic regression model for hospital survival in suPAR population with adjustment for high baseline plasma suPAR	30](#_Toc234409464)

[Table S20: Odds ratio model for hospital survival in suPAR population with addition of baseline plasma suPAR	31](#_Toc234409465)

[Figure S1: Randomisation times for the immune modulation domain (primary ferritin population).	32](#_Toc234409466)

[Figure S2: Flow diagram for ferritin analysis populations	33](#_Toc234409467)

[Figure S3: Flow diagram for suPAR analysis population	34](#_Toc234409468)

[Figure S4: Empirical distribution of organ support‐free days (OSFD) for anakinra and control in primary ferritin population ^a^	35](#_Toc234409469)

[Figure S5: Empirical distribution of organ support‐free days (OSFD) for Anakinra and control in secondary ferritin population ^a^	36](#_Toc234409470)

[Figure S6: Empirical distribution of organ support‐free days (OSFD) by ferritin quintiles in the primary ferritin population ^a^	37](#_Toc234409471)

[Figure S7a: Treatment effect of anakinra on OSFD by baseline ferritin splines in primary ferritin population	38](#_Toc234409472)

[Figure S7b: Probability that anakinra is superior to control for OSFD (OR>1) by baseline ferritin spline in primary ferritin population	38](#_Toc234409473)

[Figure S8: Forest plot showing overall anakinra effect on hospital survival in primary ferritin population with and without adjustment for ferritin	39](#_Toc234409474)

[Figure S9: Empirical distribution of organ support‐free days (OSFD) for anakinra and control in suPAR population ^a^	40](#_Toc234409475)

# Platform trial exclusion criteria

1. Death is deemed to be imminent and inevitable during the next 24 hours AND one or more of the participant, substitute decision maker or attending physician are not committed to full active treatment
2. Patient is expected to be discharged from hospital today or tomorrow
3. More than 14 days have elapsed while admitted to hospital with symptoms of an acute illness due to suspected or proven pandemic infection
4. Previous participation in this REMAP within the last 90 days

# Immune modulation domain specific exclusion criteria

1. More than 24 hours has elapsed since ICU admission
2. Patient has already received any dose of one or more of any form of interferon, anakinra, tocilizumab, or sarilumab during this hospitalization or is on long-term therapy with any of these agents prior to this hospital admission
3. Known condition or treatment resulting in ongoing immune suppression including neutropenia prior to this hospitalization
4. Patient has been randomized in a trial evaluating an immune modulation agent for proven or suspected COVID-19 infection, where the protocol of that trial requires ongoing administration of study drug
5. The treating clinician believes that participation in the domain would not be in the best interests of the patient

# Intervention-specific exclusion criteria

1. Known hypersensitivity to an agent specified as an intervention in this domain will exclude a patient from receiving that agent
2. Intention to prescribe systemic corticosteroids for any reason, other than participation in the Corticosteroid domain of this platform, will result in exclusion from receiving IFN-β1a
3. Known hypersensitivity to proteins produced by E. coli will result in exclusion from receiving anakinra
4. Known or suspected pregnancy will result in exclusion from the anakinra, IFN-β1a, tocilizumab, and sarilumab interventions. It is normal clinical practice that women admitted who are in an age group in which pregnancy is possible will have a pregnancy test conducted. The results of such tests will be used to determine interpretation of this exclusion criteria.
5. A baseline alanine aminotransferase or an aspartate aminotransferase that is more than five times the upper limit of normal will result in exclusion from receiving tocilizumab or sarilumab
6. A baseline platelet count < 50 x 109 / L will result in exclusion from receiving tocilizumab or sarilumab

# Table S1: Description of statistical models

Different models fit for the primary outcome (OSFD) are described below. The same models were also fit for survival to hospital discharge.

| **Model** **#** | **Population** | **Endpoint** | **Aim / description** | **Biomarker modelling approach** | **Covariates** | **Treatment Effects of interest** |
| --- | --- | --- | --- | --- | --- | --- |
| 1 | Primary ferritin population | OSFD | Ferritin reference model | No biomarker included | Age, sex, site, time | Overall Anakinra effect |
| 2 | Primary ferritin population | OSFD | Adjusting for Ferritin levels (as a covariate) | Ferritin (terciles) | Age, sex, site, time, baseline Ferritin terciles | Overall Anakinra effect |
| 3 | Primary ferritin population | OSFD | Ferritin as an effect modifier of anakinra effect | Ferritin (terciles) | Age, sex, site, time, baseline Ferritin terciles | Differential Anakinra effect by baseline Ferritin terciles |
| 4 | Secondary ferritin population | OSFD | Ferritin reference model; supportive analysis | No biomarker included | Age, sex, site* | Overall Anakinra effect |
| 5 | Secondary ferritin population | OSFD | Adjusting for Ferritin levels (as a covariate  ); supportive analysis | Ferritin (terciles) | Age, sex, site, baseline Ferritin terciles* | Overall Anakinra effect |
| 6 | Secondary ferritin population | OSFD | Ferritin as an effect modifier of anakinra effect; supportive analysis | Ferritin (terciles) | Age, sex, site, baseline Ferritin terciles* | Differential Anakinra effect by baseline Ferritin terciles |
| 7 | Primary ferritin population | OSFD | Adjusting for Ferritin ( covariate); supportive analysis | Ferritin (quintiles; NDLM) | Age, sex, site, time, baseline Ferritin quintiles (NDLM) | Overall Anakinra effect |
| 8 | Primary ferritin population | OSFD | Ferritin as an effect modifier of anakinra effect; supportive analysis | Ferritin (quintiles; NDLM) | Age, sex, site, time, baseline Ferritin (NDLM) | Differential Anakinra effect by baseline Ferritin (NDLM) |
| 9 | Primary ferritin population | OSFD | Adjusting for Ferritin ( covariate); supportive analysis | Ferritin (log tranformed; spline) | Age, sex, site, time, baseline (log) Ferritin (spline) | Overall Anakinra effect |
| 10 | Primary ferritin population | OSFD | Ferritin as an effect modifier of anakinra effect; supportive analysis | Ferritin (log tranformed; spline) | Age, sex, site, time, baseline (log) Ferritin (spline) | Differential Anakinra effect by baseline Ferritin (spline) |
| 11 | suPAR population | OSFD | suPAR reference model | No biomarker included | Age, sex, site, time | Overall Anakinra effect |
| 12 | suPAR population | OSFD | Adjusting for suPAR (as a covariate) | suPAR (<6 vs ≥6 subgroups) | Age, sex, site, time, baseline suPAR subgroups | Overall Anakinra effect |
| 13 | suPAR population | OSFD | suPAR as an effect modifier of anakinra | suPAR (<6 vs ≥6 subgroups) | Age, sex, site, time, baseline suPAR subgroups | Differential Anakinra effect by baseline suPAR subgroups |

*Time effects were not included in these models due to small sample sizes (most participants were recruited in the last quarter of 2020 in this population). Some of the age categories were also combined for these analyses

# Table S2: Baseline characteristics of patients in the Immune Modulation Domain of REMAP-CAP with severe COVID-19 dichotomised by availability of baseline ferritin measurement

|  | | **Patients with baseline plasma ferritin**  **(Primary ferritin population; n =1243)** | **Patients without baseline plasma ferritin**  **(n=989)** |
| --- | --- | --- | --- |
| **Treatment allocation** | |  |  |
| **Control** | | 205 (16.5) | 202 (20.4) |
| **Anakinra** | | 203 (16.3) | 167 (16.9) |
| **IL-6 receptor antagonist** | | 823 (66.2) | 612 (61.9) |
| **Interferon-β1a** | | 12 (1.0) | 8 (0.8) |
| **Age in years, median (IQR)** | | 61.0 (52.0-69.0) | 61.0 (53.0-70.0) |
| **Male sex, n (%)** | | 857 (68.9) | 688 (69.6) |
| **Race / Ethnicity ^a^, n / N (%)** | |  |  |
|  | **Asian** | 159/961 (16.5) | 118/774 (15.2) |
|  | **Black** | 40/961 (4.2) | 31/774 (4.0) |
|  | **Mixed** | 17/961 (1.8) | 10/774 (1.3) |
|  | **White** | 689/961 (71.7) | 583/774 (75.3) |
|  | **Other** | 56/961 (5.8) | 32/774 (4.1) |
| **Body-mass index ^b^, median (IQR)** | | 30.8 (26.8-35.5) (n=1152) | 30.5 (26.9-35.2) (n=867) |
| **APACHE II score ^c^, median (IQR)** | | 13.0 (8.0-19.0) (n=1229) | 13.0 (8.0-19.0) (n=972) |
| **Confirmed SARS-CoV-2 infection** | | 1065/1136 (93.8) | 845/910 (92.9) |
| **Preexisting condition ^f^, n / N (%)** | |  |  |
|  | **Diabetes** | 379 (30.5) | 297/987 (30.1) |
|  | **Respiratory disease** | 279 (22.4) | 239/987 (24.2) |
|  | **Asthma/COPD** | 236 (19.0) | 205/987 (20.8) |
|  | **Other** | 56 (4.5) | 44/987 (4.5) |
|  | **Kidney disease** | 82/1152 (7.1) | 80/898 (8.9) |
|  | **Severe cardiovascular disease** | 114/1242 (9.2) | 101/987 (10.2) |
|  | **Any immunosuppressive condition** | 35 (2.8) | 18/987 (1.8) |
|  | **Cancer** | 15 (1.2) | 8/987 (0.8) |
|  | **Chronic immunosuppressive therapy** | 14 (1.1) | 7/987 (0.7) |
|  | **Other** | 14 (1.1) | 8/987 (0.8) |
|  | **Liver cirrhosis/failure** | 2/1242 (0.2) | 5/987 (0.5) |
| **Time to enrollment, median (IQR)** | |  |  |
|  | **From hospital admission, days** | 1.3 (0.9-3.1) | 1.5 (0.9-3.5) |
|  | **From ICU admission, hours** | 13.5 (6.4-19.4) | 14.5 (7.9-19.8) |
| **Acute respiratory support, n (%)** | |  |  |
|  | **Invasive mechanical ventilation** | 410 (33.0) | 326 (33.0) |
|  | **Noninvasive ventilation only** | 511 (41.1) | 447 (45.2) |
|  | **High-flow nasal cannula** | 319 (25.7) | 215 (21.7) |
|  | **None / supplemental oxygen** | 3 (0.2) | 1 (0.1) |
|  | **ECMO** | 1/1233 (0.1) | 1/974 (0.1) |
| **Vasopressor support, n (%)** | | 235 (18.9) | 179 (18.1) |
| **PaO_2_ / FiO_2_, median (IQR)** | | 112.0 (87.0-151.0) (n=1169) | 114.0 (89.0-156.0) (n=845) |
| **Median laboratory values (IQR) ^h^** | |  |  |
|  | **C reactive protein, µg/mL** | 124.0 (70.5-197.8) (n=1090) | 124.0 (69.0-199.0) (n=708) |
|  | **D-dimer, µg/L** | 1007.0 (510.0-2412.0) (n=1017) | 806.0 (349.0-1982.8) (n=296) |
|  | **Plasma ferritin, µg/L** | 1005.0 (548.5-1688.5) | N/A |
|  | **Plasma suPAR, ng/mL** | 6.9 (5.0-8.7) (n=85) | 6.6 (5.6-8.6) (n=60) |
| **Received therapies at randomisation ^i^ – n/N (%)** | |  |  |
|  | **Corticosteroids** | 1153/1242 (92.8) | 866/988 (87.7) |
|  | **Remdesivir** | 483/1241 (38.9) | 325/987 (32.9) |
| **Country** | |  |  |
|  | **United Kingdom** | 1037 (83.4) | 880 (89.0) |
|  | **Netherlands** | 124 (10.0) | 54 (5.5) |
|  | **Saudi Arabia** | 68 (5.5) | 15 (1.5) |
|  | **Ireland** | 10 (0.8) | 27 (2.7) |
|  | **Other** | 4 (0.3) | 13 (1.3) |

Percentages may not sum to 100 because of rounding. SD denotes standard deviation.

^a^ Data collection was not approved in Canada and continental Europe. 'Other' includes 'declined' and 'multiple’. Participants (or their surrogates) self-reported their race/ ethnicity via fixed categories appropriate to their region. “Declined” does not simply represent missing data. A patient may decline to provide their race at the time of registration or the person performing the registration may decline to ask the patient to clarify race at the time of registration.

^b^ Body-mass index is the weight in kilograms divided by the square of the height in meters.

^c^ Acute Physiology and Chronic Health Evaluation II scores range 0 to 71, with higher scores indicating greater severity of illness.

^d^ Kidney disease was determined from the most recent serum creatinine level prior to this hospital admission, except in patients who were receiving dialysis. Abnormal kidney function was defined as a creatinine level of 130 μmol/L or greater (1.5mg/dL) for males or 100 μmol/L or greater (1.1 mg/dL) for females not previously receiving dialysis. Cardiovascular disease was defined as New York Heart Association class IV symptoms. Immunosuppression was defined by the receipt of recent chemotherapy, radiation, high-dose or long-term steroid treatment, or presence of immunosuppressive disease.

^e^ Laboratory results available when captured for clinical care.

^f^ Within 48hr of randomization.

# Table S3: Baseline characteristics of secondary ferritin population

|  | **Secondary ferritin population** ^a^  (N=48) |
| --- | --- |
| **Treatment allocation** |  |
| **Control** | 23 (48) |
| **Anakinra** | 25 (52) |
| **Age** (years) – Median (IQR) | 61.00 (51.5, 69.3) |
| **Age category** (years) |  |
| **<40** | 1 (2.1) |
| **40-49** | 10 (21) |
| **50-59** | 10 (21) |
| **60-69** | 15 (31) |
| **70-79** | 10 (21) |
| **80+** | 2 (4.2) |
| **Male** | 32 (67) |
| **Country** |  |
| **United Kingdom** | 27 (56) |
| **Netherlands** | 16 (33) |
| **Saudia Arabia** | 0 |
| **Other** | 5 (10.4) |
| **Time period of enrolment** |  |
| **16/01/2021 – 09/04/2021** | 0 |
| **24/10/2020 – 15/01/2021** | 27 (56) |
| **01/08/2020 – 23/10/2020** | 17 (35) |
| **09/05/2020 – 31/07/2020** | 3 (6.3) |
| **15/02/2020 – 08/05/2020** | 1 (2.1) |
| **Plasma ferritin**  (mg/L) – Median (IQR) | 1046  (679.8, 1696.0) |

a. Patients aged ≥18 years, within 24 hours of receiving respiratory or cardiovascular organ support in an intensive care unit (ICU) with suspected or microbiologically confirmed COVID-19 who were eligible for both anakinra and control within the COVID-19 immune modulation domain of REMAP-CAP and had available baseline plasma ferritin results.

# Table S4: Outcomes of secondary ferritin population

|  | **Secondary ferritin population** ^a^  (N=48) |
| --- | --- |
| **OSFD** – median (IQR)  **Overall population**  **By treatment group:**  **Control**  **Anakinra** | 9.5 (-1.0, 18.0)  10.0 (-1.0, 17.0)  9.0 (-1.0, 18.0) |
| **In-hospital mortality** – N(%)  **Overall population**  **By treatment group:**  **Control**  **Anakinra** | 16 (33.3)  7 (30.4)   9 (36.0) |

a. Patients aged ≥18 years, within 24 hours of receiving respiratory or cardiovascular organ support in an intensive care unit (ICU) with suspected or microbiologically confirmed COVID-19 who were eligible for both anakinra and control within the COVID-19 immune modulation domain of REMAP-CAP and had available baseline plasma ferritin results.

# Table S5: OSFDs stratified by ferritin tercile

|  | **Primary ferritin population** ^a,b^ | **Secondary ferritin population** ^a^**^,c^** |
| --- | --- | --- |
| **Ferritin tercile 1** |  | |
| **Overall population** | 10.0 (-1.0, 17.0) | 13.0 (-1.0, 17.0) |
| **Control** | 9.0 (-1.0, 16.0) | 6.5 (-1.0, 17.2) |
| **Anakinra** | 7.5 (-1.0, 17.0) | 17.0 (-1.0, 17.0) |
| **IL-6 receptor antagonist** | 11.0 (-1.0, 18.0) | N/a |
| **Interferon-β1a** | 7.0 (0.0, 10.0) | N/a |
| **Ferritin tercile 2** |  | |
| **Overall population** | 4.0 (-1.0, 16.0) | 13.0 (0.5, 18.0) |
| **Control** | 4.0 (-1.0, 16.0) | 11.5 (4.2, 17.0) |
| **Anakinra** | 1.0 (-1.0, 17.0) | 14.0 (-1.0, 18.0) |
| **IL-6 receptor antagonist** | 5.0 (-1.0, 16.0) | N/a |
| **Interferon-β1a** | 0.0 (0.0, 0.0) | N/a |
| **Ferritin tercile 3** |  | |
| **Overall population** | 0.0 (-1.0, 15.0) | 0.0 (-1.0, 15.0) |
| **Control** | 0.0 (-1.0, 13.0) | 0.0 (-1.0, 14.0) |
| **Anakinra** | 0.0 (-1.0, 10.0) | 0.0 (-1.0, 16.5) |
| **IL-6 receptor antagonist** | 3.0 (-1.0, 16.0) | N/a |
| **Interferon-β1a** | 5.5 (-0.8, 15.5) | N/a |

Data presented as median (IQR).

Ferritin tercile 1: Primary ferritin population n=413; secondary ferritin population n=13

Ferritin tercile 2: Primary ferritin population n=415; secondary ferritin population n=19

Ferritin tercile 3: Primary ferritin population n=415; secondary ferritin population n=16

a. Patients aged ≥18 years, within 24 hours of receiving respiratory or cardiovascular organ support in an intensive care unit (ICU) with suspected or microbiologically confirmed COVID-19 who were
b. recruited to the COVID-19 immune modulation domain of REMAP-CAP and whom had available baseline plasma ferritin results.
c. eligible for both anakinra and control within the COVID-19 immune modulation domain of REMAP-CAP and had available baseline plasma ferritin results.

# Table S6: In-hospital mortality stratified by ferritin tercile

|  | **Primary ferritin population** ^a,b^ | **Secondary ferritin population** ^a^**^,c^** |
| --- | --- | --- |
| **Ferritin tercile 1** |  | |
| **Overall population** | 121 (29.3) | 5 (38.5) |
| **Control** | 28 (32.9) | 3 (37.5) |
| **Anakinra** | 24 (34.3) | 2 (40.0) |
| **IL-6 receptor antagonist** | 68 (26.9) | N/a |
| **Interferon-β1a** | 1 (20.0) | N/a |
| **Ferritin tercile 2** |  | |
| **Overall population** | 139 (33.5) | 5 (26.3) |
| **Control** | 22 (35.5) | 2 (20.0) |
| **Anakinra** | 21 (31.8) | 3 (33.3) |
| **IL-6 receptor antagonist** | 96 (33.6) | N/a |
| **Interferon-β1a** | 0 (0) | N/a |
| **Ferritin tercile 3** |  | |
| **Overall population** | 157 (37.8) | 6 (37.5) |
| **Control** | 22 (37.9) | 2 (40.0) |
| **Anakinra** | 32 (47.8) | 4 (36.4) |
| **IL-6 receptor antagonist** | 101 (35.6) | N/a |
| **Interferon-β1a** | 2 (33.3) | N/a |

Data presented as N(%).

Ferritin tercile 1: Primary ferritin population n=413; secondary ferritin population n=13

Ferritin tercile 2: Primary ferritin population n=415; secondary ferritin population n=19

Ferritin tercile 3: Primary ferritin population n=415; secondary ferritin population n=16

a. Patients aged ≥18 years, within 24 hours of receiving respiratory or cardiovascular organ support in an intensive care unit (ICU) with suspected or microbiologically confirmed COVID-19 who were
b. recruited to the COVID-19 immune modulation domain of REMAP-CAP and whom had available baseline plasma ferritin results.
c. eligible for both anakinra and control within the COVID-19 immune modulation domain of REMAP-CAP and had available baseline plasma ferritin results.

# Table S7: Cumulative logistic regression model for OSFD in primary ferritin population (Reference model)

| **Variable** | **Median OR** | **CrI** | **Mean OR** | **SD** | **Pr(OR>1)** |
| --- | --- | --- | --- | --- | --- |
| Anakinra | 1.01 | (0.68, 1.50) | 1.03 | 0.21 | 0.521 |
| Age<39 | 4.83 | (3.18, 7.38) | 4.94 | 1.08 |  |
| Age 40-49 | 2.42 | (1.77, 3.32) | 2.45 | 0.40 |  |
| Age 50-59 | 2.22 | (1.70, 2.90) | 2.24 | 0.31 |  |
| Age 70-79 | 0.54 | (0.39, 0.73) | 0.54 | 0.09 |  |
| Age 80+ | 0.34 | (0.18, 0.62) | 0.35 | 0.11 |  |
| Female | 1.37 | (1.10, 1.70) | 1.38 | 0.15 |  |

Multivariable analysis of patients aged ≥18 years, within 24 hours of receiving respiratory or cardiovascular organ support in an intensive care unit (ICU) with suspected or microbiologically confirmed COVID-19 who were recruited to the COVID-19 immune modulation domain of REMAP-CAP and whom had available baseline plasma ferritin results

# Table S8: Cumulative logistic regression model for OSFD with ferritin quintiles-anakinra interaction terms in primary ferritin population

| **Variable** | **Median OR** | **CrI** | **Mean OR** | **SD** | **Pr(OR>1)** |
| --- | --- | --- | --- | --- | --- |
| Anakinra (Ferritin Quintile 1) | 1.10 | (0.67, 1.89) | 1.15 | 0.31 | 0.650 |
| Anakinra (Ferritin Quintile 2) | 1.15 | (0.73, 1.93) | 1.20 | 0.31 | 0.723 |
| Anakinra (Ferritin Quintile 3) | 1.03 | (0.65, 1.67) | 1.07 | 0.26 | 0.557 |
| Anakinra (Ferritin Quintile 4) | 0.90 | (0.51, 1.44) | 0.92 | 0.24 | 0.337 |
| Anakinra (Ferritin Quintile 5) | 0.89 | (0.50, 1.47) | 0.91 | 0.25 | 0.331 |
| Age<39 | 4.84 | (3.18, 7.42) | 4.96 | 1.08 |  |
| Age 40-49 | 2.41 | (1.75, 3.31) | 2.44 | 0.40 |  |
| Age 50-59 | 2.24 | (1.72, 2.93) | 2.26 | 0.31 |  |
| Age 70-79 | 0.55 | (0.41, 0.75) | 0.56 | 0.09 |  |
| Age 80+ | 0.34 | (0.18, 0.63) | 0.36 | 0.12 |  |
| Female | 1.25 | (1.00, 1.58) | 1.26 | 0.15 |  |

Multivariable analysis of patients aged ≥18 years, within 24 hours of receiving respiratory or cardiovascular organ support in an intensive care unit (ICU) with suspected or microbiologically confirmed COVID-19 who were recruited to the COVID-19 immune modulation domain of REMAP-CAP and whom had available baseline plasma ferritin results

# Table S9: Cumulative logistic regression model for OSFD in secondary ferritin population (reference model)

| **Variable** | **Median OR** | **CrI** | **Mean OR** | **SD** | **Pr(OR>1)** |
| --- | --- | --- | --- | --- | --- |
| Anakinra | 1.14 | (0.43, 3.03) | 1.30 | 0.69 | 0.607 |
| Age<=49 | 2.85 | (0.92, 9.09) | 3.38 | 2.15 |  |
| Age 50-59 | 1.78 | (0.59, 5.40) | 2.11 | 1.32 |  |
| Age 70+ | 1.45 | (0.42, 4.96) | 1.76 | 1.24 |  |
| Female | 0.36 | (0.13, 0.99) | 0.41 | 0.23 |  |

Multivariable analysis of patients aged ≥18 years, within 24 hours of receiving respiratory or cardiovascular organ support in an intensive care unit (ICU) with suspected or microbiologically confirmed COVID-19 who were recruited to the COVID-19 immune modulation domain of REMAP-CAP and whom had available baseline plasma ferritin results

# Table S10: Cumulative logistic regression model for OSFD with ferritin terciles as covariates in secondary ferritin population

| **Variable** | **Median OR** | **CrI** | **Mean OR** | **SD** | **Pr(OR>1)** |
| --- | --- | --- | --- | --- | --- |
| Anakinra | 1.21 | (0.45, 3.27) | 1.38 | 0.75 | 0.646 |
| Baseline Ferritin Tercile 2 | 1.61 | (0.55, 4.70) | 1.86 | 1.11 | NA |
| Baseline Ferritin Tercile 3 | 0.65 | (0.20, 2.03) | 0.77 | 0.50 | NA |
| Age<=49 | 2.72 | (0.87, 8.48) | 3.22 | 2.05 | NA |
| Age 50-59 | 1.78 | (0.57, 5.64) | 2.12 | 1.35 | NA |
| Age 70+ | 1.60 | (0.45, 5.58) | 1.97 | 1.41 | NA |
| Female | 0.30 | (0.10, 0.88) | 0.35 | 0.21 | NA |

Multivariable analysis in patients aged ≥18 years, within 24 hours of receiving respiratory or cardiovascular organ support in an intensive care unit (ICU) with suspected or microbiologically confirmed COVID-19 who were eligible for both anakinra and control within the COVID-19 immune modulation domain of REMAP-CAP and had available baseline plasma ferritin results.

# Table S11: Cumulative logistic regression model for OSFD with ferritin terciles-anakinra interaction terms in secondary ferritin population

| **Variable** | **Median OR** | **CrI** | **Mean OR** | **SD** | **Pr(OR>1)** |
| --- | --- | --- | --- | --- | --- |
| Anakinra (Ferritin Tercile 1) | 1.71 | (0.39, 7.60) | 2.27 | 1.99 | 0.760 |
| Anakinra (Ferritin Tercile 2) | 0.97 | (0.27, 3.49) | 1.21 | 0.88 | 0.483 |
| Anakinra (Ferritin Tercile 3) | 0.96 | (0.25, 3.61) | 1.21 | 0.93 | 0.475 |
| Baseline Ferritin Tercile 2 | 1.80 | (0.56, 5.79) | 2.15 | 1.40 |  |
| Baseline Ferritin Tercile 3 | 0.72 | (0.20, 2.60) | 0.89 | 0.65 |  |
| Age<=49 | 2.68 | (0.85, 8.41) | 3.17 | 2.04 |  |
| Age 50-59 | 1.81 | (0.58, 5.63) | 2.13 | 1.34 |  |
| Age 70+ | 1.68 | (0.47, 5.82) | 2.06 | 1.46 |  |
| Female | 0.27 | (0.09, 0.82) | 0.32 | 0.19 |  |

Multivariable analysis of patients aged ≥18 years, within 24 hours of receiving respiratory or cardiovascular organ support in an intensive care unit (ICU) with suspected or microbiologically confirmed COVID-19 who were eligible for both anakinra and control within the COVID-19 immune modulation domain of REMAP-CAP and had available baseline plasma ferritin results.

# Table S12: Logistic regression model for hospital survival exploring differential treatment effect with ferritin terciles in secondary ferritin population

| **Variable** | **Median OR** | **CrI** | **Mean OR** | **SD** | **Pr(OR>1)** |
| --- | --- | --- | --- | --- | --- |
| Anakinra (Ferritin Tercile 1) | 1.15 | (0.25, 5.46) | 1.57 | 1.50 | 0.569 |
| Anakinra (Ferritin Tercile 2) | 0.89 | (0.21, 3.88) | 1.18 | 1.04 | 0.436 |
| Anakinra (Ferritin Tercile 3) | 0.98 | (0.23, 4.19) | 1.29 | 1.11 | 0.487 |
| Baseline Ferritin Tercile 2 | 1.66 | (0.43, 6.59) | 2.12 | 1.68 |  |
| Baseline Ferritin Tercile 3 | 0.84 | (0.21, 3.42) | 1.08 | 0.89 |  |
| Age<=49 | 4.01 | (1.02, 17.07) | 5.26 | 4.49 |  |
| Age 50-59 | 2.60 | (0.68, 10.86) | 3.39 | 2.86 |  |
| Age 70+ | 0.89 | (0.23, 3.36) | 1.12 | 0.85 |  |
| Female | 0.37 | (0.11, 1.27) | 0.45 | 0.32 |  |

Multivariable analysis of patients aged ≥18 years, within 24 hours of receiving respiratory or cardiovascular organ support in an intensive care unit (ICU) with suspected or microbiologically confirmed COVID-19 who were eligible for both anakinra and control within the COVID-19 immune modulation domain of REMAP-CAP and had available baseline plasma ferritin results.

# Table S13: Baseline characteristics based on availability of baseline plasma suPAR

|  | | | **Patients with available plasma suPAR**  (suPAR population; n=145) | **Patients without baseline suPAR who were randomized to control or anakinra**  (n=632) |
| --- | --- | --- | --- | --- |
| **Treatment allocation** | | |  |  |
| **Control** | | | 78 (53.8) | 329 (52.1) |
| **Anakinra** | | | 67 (46.2) | 303 (47.9) |
| **Age in years, median (IQR)** | | | 61.0 (53.0-70.0) | 61.5 (53.0-69.0) |
| **Male sex, n (%)** | | | 106 (73.1) | 445 (70.4) |
| **Race / Ethnicity ^a^, n / N (%)** | | |  |  |
|  | **Asian** | | 25/133 (18.8) | 68/420 (16.2) |
|  | **Black** | | 7/133 (5.3) | 15/420 (3.6) |
|  | **Mixed** | | 2/133 (1.5) | 11/420 (2.6) |
|  | **White** | | 93/133 (69.9) | 303/420 (72.1) |
|  | **Other** | | 6/133 (4.5) | 23/420 (5.5) |
| **Body-mass index ^b^, median (IQR)** | | | 31.2 (27.1-34.3) (n=127) | 30.3 (26.7-35.3) (n=589) |
| **APACHE II score ^c^, median (IQR)** | | | 12.0 (7.0-19.2) (n=144) | 13.0 (8.0-19.0) (n=621) |
| **Confirmed SARS-CoV-2 infection** | | | 136/140 (97.1) | 531/579 (91.7) |
| **Preexisting condition ^d^, n / N (%)** | | |  |  |
|  | | **Diabetes** | 51 (35.2) | 226 (35.8) |
|  | **Respiratory disease** | | 27 (18.6) | 154 (24.4) |
|  | **Asthma/COPD** | | 24 (16.6) | 133 (21.0) |
|  | **Other** | | 4 (2.8) | 29 (4.6) |
|  | **Kidney disease** | | 10/132 (7.6) | 57/588 (9.7) |
|  | **Severe cardiovascular disease** | | 17/144 (11.8) | 71 (11.2) |
|  | **Any immunosuppressive condition** | | 4 (2.8) | 17 (2.7) |
|  | **Cancer** | | 2 (1.4) | 11 (1.7) |
|  | **Chronic immunosuppressive therapy** | | 0 (0.0) | 7 (1.1) |
|  | **Other** | | 2 (1.4) | 4 (0.6) |
|  | **Liver cirrhosis/failure** | | 0/144 (0.0) | 3 (0.5) |
| **Time to enrollment, median (IQR)** | | |  |  |
|  | **From hospital admission, days** | | 1.1 (0.8-2.6) | 1.4 (0.9-3.2) |
|  | **From ICU admission, hours** | | 15.0 (7.5-20.1) | 13.7 (6.8-19.4) |
| **Acute respiratory support, n (%)** | | |  |  |
|  | **Invasive mechanical ventilation** | | 42 (29.0) | 218 (34.5) |
|  | **Noninvasive ventilation only** | | 68 (46.9) | 234 (37.0) |
|  | **High-flow nasal cannula** | | 33 (22.8) | 179 (28.3) |
|  | **None / supplemental oxygen** | | 2 (1.4) | 1 (0.2) |
|  | **ECMO** | | 1 (0.7) | 0/623 (0.0) |
| **Vasopressor support, n (%)** | | | 26 (17.9) | 130 (20.6) |
| **PaO_2_ / FiO_2_, median (IQR)** | | | 111.5 (88.8-169.2) (n=128) | 114.0 (86.0-157.0) (n=562) |
| **Median laboratory values (IQR)** ^e^ | | |  |  |
|  | **C reactive protein, µg/mL** | | 124.0 (77.0-206.0) (n=117) | 118.0 (67.8-193.5) (n=464) |
|  | **D-dimer, µg/L** | | 1006.5 (474.5-1620.0) (n=82) | 1000.0 (454.8-2339.5) (n=350) |
|  | **Plasma ferritin, µg/L** | | 914.0 (458.0-1787.0) (n=85) | 908.0 (487.0-1652.0) (n=323) |
|  | **Plasma suPAR, ng/mL** | | 6.7 (5.3-8.7) | N/A |
| **Received therapies at randomisation** ^f^ **– n/N (%)** | | |  |  |
|  | **Corticosteroids** | | 136 (93.8) | 533/631 (84.5) |
|  | **Remdesivir** | | 75 (51.7) | 195/630 (31.0) |
| **Country** | | |  |  |
|  | **United Kingdom** | | 145 (100.0) | 461 (72.9) |
|  | **Netherlands** | | 0 (0.0) | 92 (14.6) |
|  | **Saudi Arabia** | | 0 (0.0) | 42 (6.6) |
|  | **Ireland** | | 0 (0.0) | 24 (3.8) |
|  | **Other** | | 0 (0.0) | 13 (2.1) |

Percentages may not sum to 100 because of rounding. SD denotes standard deviation.

^a^ Data collection was not approved in Canada and continental Europe. 'Other' includes 'declined' and 'multiple’. Participants (or their surrogates) self-reported their race/ ethnicity via fixed categories appropriate to their region. “Declined” does not simply represent missing data. A patient may decline to provide their race at the time of registration or the person performing the registration may decline to ask the patient to clarify race at the time of registration.

^b^ Body-mass index is the weight in kilograms divided by the square of the height in meters.

^c^ Acute Physiology and Chronic Health Evaluation II scores range 0 to 71, with higher scores indicating greater severity of illness.

^d^ Kidney disease was determined from the most recent serum creatinine level prior to this hospital admission, except in patients who were receiving dialysis. Abnormal kidney function was defined as a creatinine level of 130 μmol/L or greater (1.5mg/dL) for males or 100 μmol/L or greater (1.1 mg/dL) for females not previously receiving dialysis. Cardiovascular disease was defined as New York Heart Association class IV symptoms. Immunosuppression was defined by the receipt of recent chemotherapy, radiation, high-dose or long-term steroid treatment, or presence of immunosuppressive disease.

^e^ Laboratory results available when captured for clinical care.

^f^ Within 48hr of randomization.

# Table S14: Baseline characteristics in suPAR population dichotomised by baseline plasma suPAR

|  | **Low suPAR**  (<6 ng/ml; n=60) | **High suPAR**  (≥6 ng/ml; n=85) |
| --- | --- | --- |
| **Treatment allocation** |  |  |
| **Control** | 46 (77) | 32 (38) |
| **Anakinra** | 14 (23) | 53 (62) |
| **Age category** (years) |  |  |
| **<40** | 6 (10) | 1 (1.2) |
| **40-49** | 10 (17) | 13 (15) |
| **50-59** | 18 (30) | 18 (21) |
| **60-69** | 13 (22) | 27 (32) |
| **70-79** | 12 (20) | 20 (24) |
| **80+** | 1 (1.7) | 6 (7.1) |
| **Male** | 39 (65) | 67 (79) |
| **Country** |  |  |
| **United Kingdom** | 60 (100) | 85 (100) |
| **Time period of enrolment** |  |  |
| **09/05/2020 – 31/07/2020** | 9 (15) | 4 (4.7) |
| **01/08/2020 – 23/10/2020** | 15 (25) | 17 (20) |
| **24/10/2020 – 15/01/2021** | 36 (60) | 64 (75) |
| **Plasma suPAR**  (ng / ml) – Median (IQR) | 4.99  (4.29, 5.57) | 8.30  (7.09, 10.09) |

Patients aged ≥18 years, within 24 hours of receiving respiratory or cardiovascular organ support in an intensive care unit (ICU) with suspected or microbiologically confirmed COVID-19 who were randomised to anakinra or control within the COVID-19 immune modulation domain of REMAP-CAP and whom had available baseline plasma suPAR results

# Table S15: OSFD and hospital mortality in suPAR population dichotomised by baseline plasma suPAR

|  | **High plasma suPAR**  (≥6 ng/ml; n=85) | **Low plasma suPAR**  (<6 ng/ml; n=60) |
| --- | --- | --- |
| **OSFD** (median [IQR]) | -1.0 [-1.0, 9.0] | 13.0 [-1.0, 17.0] |
| **In-hospital mortality** – N(%) | 46 (54.1) | 16 (26.7) |

Patients aged ≥18 years, within 24 hours of receiving respiratory or cardiovascular organ support in an intensive care unit (ICU) with suspected or microbiologically confirmed COVID-19 who were randomised to anakinra or control within the COVID-19 immune modulation domain of REMAP-CAP and whom had available baseline plasma suPAR results

# Table S16: Cumulative logistic regression model for OSFD in suPAR population (reference model)

| **Variable** | **Median OR** | **CrI** | **Mean OR** | **SD** | **Pr(OR>1)** |
| --- | --- | --- | --- | --- | --- |
| Anakinra | 0.92 | (0.48, 1.76) | 0.98 | 0.33 | 0.407 |
| Age<39 | 8.18 | (2.51, 27.48) | 9.92 | 6.80 |  |
| Age 40-49 | 1.96 | (0.85, 4.55) | 2.15 | 0.97 |  |
| Age 50-59 | 2.07 | (0.99, 4.34) | 2.23 | 0.87 |  |
| Age 70-79 | 0.60 | (0.26, 1.36) | 0.66 | 0.29 |  |
| Age 80+ | 0.48 | (0.12, 1.66) | 0.58 | 0.42 |  |
| Female | 1.15 | (0.58, 2.21) | 1.21 | 0.42 |  |

Multivariable regression of patients aged ≥18 years, within 24 hours of receiving respiratory or cardiovascular organ support in an intensive care unit (ICU) with suspected or microbiologically confirmed COVID-19 who were randomised to anakinra or control within the COVID-19 immune modulation domain of REMAP-CAP and whom had available baseline plasma suPAR results

# Table S17: Cumulative logistic regression model for OSFDs in suPAR analysis population with adjustment for high baseline plasma suPAR

| **Variable** | **Median odds ratio**  [95% Credible Interval] | **Mean odds ratio**  (SD) | **Pr(OR>1)** |
| --- | --- | --- | --- |
| **Anakinra** | 1.33 [0.66, 2.61] | 1.41 (0.51) | 0.789 |
| **High baseline plasma suPAR**  (≥6 ng/ml) | 0.36 [0.18, 0.72] | 0.39 (0.14) |  |
| **Age<39** | 6.74 [2.05, 23.02] | 8.21 (5.68) |  |
| **Age 40-49** | 1.81 [0.78, 4.14] | 1.98 (0.89) |  |
| **Age 50-59** | 1.83 [0.87, 3.86] | 1.97 (0.78) |  |
| **Age 70-79** | 0.59 [0.25, 1.33] | 0.64 (0.28) |  |
| **Age 80+** | 0.55 [0.14, 1.97] | 0.68 (0.49) |  |
| **Female** | 1.11 [0.56, 2.15] | 1.18 (0.41) |  |

Multivariable (cumulative logistic) regression of patients aged ≥18 years, within 24 hours of receiving respiratory or cardiovascular organ support in an intensive care unit (ICU) with suspected or microbiologically confirmed COVID-19 who were randomised to anakinra or control within the COVID-19 immune modulation domain of REMAP-CAP and whom had available baseline plasma suPAR results

# Table S18: Logistic regression model for hospital survival in suPAR population (reference model)

| **Variable** | **Median OR** | **CrI** | **Mean OR** | **SD** | **Pr(OR>1)** |
| --- | --- | --- | --- | --- | --- |
| Anakinra | 0.60 | (0.29, 1.24) | 0.64 | 0.25 | 0.087 |
| Age<39 | 9.06 | (1.98, 49.56) | 13.17 | 14.36 |  |
| Age 40-49 | 2.47 | (0.97, 6.49) | 2.79 | 1.48 |  |
| Age 50-59 | 3.38 | (1.41, 8.48) | 3.79 | 1.88 |  |
| Age 70-79 | 0.62 | (0.25, 1.47) | 0.68 | 0.32 |  |
| Age 80+ | 0.48 | (0.12, 1.75) | 0.59 | 0.44 |  |
| Female | 1.25 | (0.55, 2.82) | 1.36 | 0.59 |  |

Multivariable (logistic) regression of patients aged ≥18 years, within 24 hours of receiving respiratory or cardiovascular organ support in an intensive care unit (ICU) with suspected or microbiologically confirmed COVID-19 who were randomised to anakinra or control within the COVID-19 immune modulation domain of REMAP-CAP and whom had available baseline plasma suPAR results

# Table S19: Logistic regression model for hospital survival in suPAR population with adjustment for high baseline plasma suPAR

| **Variable** | **Median** | **CrI** | **Mean** | **SD** | **Pr(OR>1)** |
| --- | --- | --- | --- | --- | --- |
| Anakinra | 0.78 | (0.35, 1.71) | 0.85 | 0.35 | 0.269 |
| High baseline plasma suPAR  (≥6 ng/ml) | 0.45 | (0.20, 0.97) | 0.48 | 0.20 |  |
| Age<39 | 8.31 | (1.77, 46.41) | 12.14 | 13.41 |  |
| Age 40-49 | 2.31 | (0.90, 6.24) | 2.63 | 1.41 |  |
| Age 50-59 | 3.24 | (1.35, 8.16) | 3.62 | 1.81 |  |
| Age 70-79 | 0.61 | (0.25, 1.45) | 0.67 | 0.31 |  |
| Age 80+ | 0.53 | (0.13, 1.94) | 0.66 | 0.49 |  |
| Female | 1.15 | (0.51, 2.65) | 1.26 | 0.56 |  |

Multivariable (logistic) regression of patients aged ≥18 years, within 24 hours of receiving respiratory or cardiovascular organ support in an intensive care unit (ICU) with suspected or microbiologically confirmed COVID-19 who were randomised to anakinra or control within the COVID-19 immune modulation domain of REMAP-CAP and whom had available baseline plasma suPAR results

# Table S20: Odds ratio model for hospital survival in suPAR population with addition of baseline plasma suPAR

| **Variable** | **Median OR** | **CrI** | **Mean OR** | **SD** | **Pr(OR>1)** |
| --- | --- | --- | --- | --- | --- |
| Anakinra (low baseline plasma suPAR) | 1.39 | (0.43, 4.84) | 1.71 | 1.21 | 0.704 |
| Anakinra (high baseline plasma suPAR) | 0.61 | (0.25, 1.46) | 0.67 | 0.32 | 0.136 |
| High baseline plasma suPAR (≥6) | 0.57 | (0.23, 1.38) | 0.63 | 0.30 |  |
| Age<39 | 8.37 | (1.78, 46.60) | 12.17 | 13.26 |  |
| Age 40-49 | 2.48 | (0.95, 6.67) | 2.82 | 1.52 |  |
| Age 50-59 | 3.27 | (1.34, 8.21) | 3.65 | 1.83 |  |
| Age 70-79 | 0.60 | (0.25, 1.45) | 0.67 | 0.32 |  |
| Age 80+ | 0.55 | (0.13, 2.03) | 0.68 | 0.52 |  |
| Female | 1.14 | (0.49, 2.62) | 1.25 | 0.55 |  |

Multivariable (logistic) regression of patients aged ≥18 years, within 24 hours of receiving respiratory or cardiovascular organ support in an intensive care unit (ICU) with suspected or microbiologically confirmed COVID-19 who were randomised to anakinra or control within the COVID-19 immune modulation domain of REMAP-CAP and whom had available baseline plasma suPAR results

# Figure S1: Randomisation times for the immune modulation domain (primary ferritin population).

# Figure S2: Flow diagram for ferritin analysis populations

# Figure S3: Flow diagram for suPAR analysis population

# Figure S4: Empirical distribution of organ support‐free days (OSFD) for anakinra and control in primary ferritin population ^a^

a. Patients aged ≥18 years, within 24 hours of receiving respiratory or cardiovascular organ support in an intensive care unit (ICU) with suspected or microbiologically confirmed COVID-19 who were recruited to the COVID-19 immune modulation domain of REMAP-CAP and whom had available baseline plasma ferritin results

# Figure S5: Empirical distribution of organ support‐free days (OSFD) for Anakinra and control in secondary ferritin population ^a^

a. Patients aged ≥18 years, within 24 hours of receiving respiratory or cardiovascular organ support in an intensive care unit (ICU) with suspected or microbiologically confirmed COVID-19 who were eligible for both anakinra and control within the COVID-19 immune modulation domain of REMAP-CAP and had available baseline plasma ferritin results.

# Figure S6: Empirical distribution of organ support‐free days (OSFD) by ferritin quintiles in the primary ferritin population ^a^

a. Patients aged ≥18 years, within 24 hours of receiving respiratory or cardiovascular organ support in an intensive care unit (ICU) with suspected or microbiologically confirmed COVID-19 who were recruited to the COVID-19 immune modulation domain of REMAP-CAP and whom had available baseline plasma ferritin results

# Figure S7a: Treatment effect of anakinra on OSFD by baseline ferritin splines in primary ferritin population

# Figure S7b: Probability that anakinra is superior to control for OSFD (OR>1) by baseline ferritin spline in primary ferritin population

# Figure S8: Forest plot showing overall anakinra effect on hospital survival in primary ferritin population with and without adjustment for ferritin

# Figure S9: Empirical distribution of organ support‐free days (OSFD) for anakinra and control in suPAR population ^a^

a. Patients aged ≥18 years, within 24 hours of receiving respiratory or cardiovascular organ support in an intensive care unit (ICU) with suspected or microbiologically confirmed COVID-19 who were randomised to anakinra or control within the COVID-19 immune modulation domain of REMAP-CAP and whom had available baseline plasma suPAR results.
